# Diffusion Entropy vs. Multiscale and Rényi Entropy to Detect Progression of Autonomic Neuropathy

**DOI:** 10.3389/fphys.2020.607324

**Published:** 2021-01-14

**Authors:** Herbert F. Jelinek, Rohisha Tuladhar, Garland Culbreth, Gyanendra Bohara, David Cornforth, Bruce. J. West, Paolo Grigolini

**Affiliations:** ^1^Health Engineering Innovation Center, Khalifa University, Abu Dhabi, United Arab Emirates; ^2^Department of Biomedical Engineering, Khalifa University, Abu Dhabi, United Arab Emirates; ^3^Department of Biology, The University of Texas at San Antonio, San Antonio, TX, United States; ^4^Center for Nonlinear Science, The University of North Texas, Denton, TX, United States; ^5^The University of Texas Southwestern Medical Center, Dallas, TX, United States; ^6^Applied Informatics Research Group, Faculty of Science and IT, The University of Newcastle, Callaghan, NSW, Australia; ^7^Office of the Director, Army Research Office, Research Triangle Park, Durham, NC, United States

**Keywords:** crucial events, complexity, diffusion entropy, multiscale entropy, Rényi entropy, autonomic neuropathy

## Abstract

We review the literature to argue the importance of the occurrence of crucial events in the dynamics of physiological processes. Crucial events are interpreted as short time intervals of turbulence, and the time distance between two consecutive crucial events is a waiting time distribution density with an inverse power law (IPL) index μ, with μ < 3 generating non-stationary behavior. The non-stationary condition is characterized by two regimes of the IPL index: (a) perennial non-stationarity, with 1 < μ < 2 and (b) slow evolution toward the stationary regime, with 2 < μ < 3. Human heartbeats and brain dynamics belong to the latter regime, with healthy physiological processes tending to be closer to the border with the perennial non-stationary regime with μ = 2. The complexity of cognitive tasks is associated with the mental effort required to address a difficult task, which leads to an increase of μ with increasing task difficulty. On this basis we explore the conjecture that disease evolution leads the IPL index μ moving from the healthy condition μ = 2 toward the border with Gaussian statistics with μ = 3, as the disease progresses. Examining heart rate time series of patients affected by diabetes-induced autonomic neuropathy of varying severity, we find that the progression of cardiac autonomic neuropathy (CAN) indeed shifts μ from the border with perennial variability, μ = 2, to the border with Gaussian statistics, μ = 3 and provides a novel, sensitive index for assessing disease progression. We find that at the Gaussian border, the dynamical complexity of crucial events is replaced by Gaussian fluctuation with long-time memory.

## 1. Introduction

Heart rate analysis and specifically heart rate variability (HRV) analysis has proven to be a useful adjunct feature for clinical medicine (Javorka et al., 2008; Huikuri et al., 2009; Hu et al., 2010; Lake, 2011). However, obtaining results that allow classification of pathology or determining risk of morbidity or mortality are only one part of the solution. Descriptive features sensitive to characteristics of a time series need also to be understood in terms of their explanatory power of the process they are measuring. Herein, we address the latter point.

Entropy-derived measures including Sample Entropy, Multiscale Entropy (MSE), and Rényi Entropy have been applied for early identification of sepsis and analysis of diabetes and cardiovascular diseases (CVD) (Oida et al., 1999; Lake et al., 2002; Costa and Healy, 2003; Lake, 2006; Valencia et al., 2009; Cornforth et al., 2015; Kohnert et al., 2018; Jelinek et al., 2019). Applying MSE (*S*_*E*_) (Costa et al., 2002, 2005) turned out to be a very efficient technique for the analysis of HRV and in identifying cardiac pathology. MSE bypasses the computational difficulties to evaluate the Kolmogorov-Sinai (KS) entropy (Costa et al., 2002; Allegrini et al., 2003). Applying a coarse-graining procedure to the experimental time series of the interbeat fluctuations (RR intervals) provides a mathematical model to describe the bio-signal associated with heart rate fluctuations.

Hereby we also adopt the research directions proposed in Allegrini et al. (2003) to address the problem of the connection between entropy and 1/*f* noise. Moving in the directions of Allegrini et al. (2003) allows us to address the Chialvo challenge (Chialvo, 2002) to propose a robust model describing a better understanding of the dynamics of heart rate time series and of physiological processes in general. This model rests on self-organized criticality. Self-organized criticality can be used to advocate a model generating crucial events, interpreted as short-time turbulent intervals. We must stress that the main conclusion of the present manuscript, as we shall see hereby, is that the crucial events are important in the first phase of cardiac autonomic neuropathy (early CAN). In the final definite CAN condition crucial events are replaced by a source of complexity generated by a stationary but not integrable correlation function. The model to use in this case, as discussed in Bologna et al. (2013), should be a model explaining why the two sources of complexity may both explain the signal characteristics. We plan to devote further research work to further build up this model.

The Rényi entropy was shown by Cornforth et al. (2014) to be a valuable tool in the study of Cardiac Autonomic Neuropathy (CAN). These authors, using Rényi Rényi entropy with α < 0 were able to divide a patient cohort into three distinct groups, normal, early, and definite CAN. Rényi entropy was proposed as an extension of multiscale entropy (MSE) and overcame the shortcomings of *S*_*E*_ by addressing the boundary problem inherent in the histogram method of *S*_*E*_.

In this new approach a density measure can be calculated for the individual RR interval using a Gaussian approach. A Gaussian kernel is centered on the individual RR interval and all RR intervals are added, weighted by the Gaussian function, based on the distance between the individual RR intervals. Apart from the advantage of allowing a continuous rather than a discretised measure, this method also allows more than one dimension, as long as a suitable distance measure can be provided. In the case of RR intervals, higher dimensions allow sequences of RR intervals to be compared, rather than relying solely on individual RR intervals. In the case of Rényi entropy, we actually calculate the probability of each sequence. This differs from MSE, where we count the number of sequences that are unique, so that this is a more approximate way of calculating the PDF for each sequence.

In this paper, we find a method to determine the role of crucial events in generating the healthy RR fluctuations. Following Shuster (1988) we define crucial events using the concept of intermittent turbulence. Intermittent turbulence is a concept often applied to describe and model complex physical behaviors that can be described as fractal. Intermittency of any physical phenomena such as turbulence in fluid flow or changes in heart rate changes the statistical properties of the scaling laws for the different moments of velocity, energy distribution, and diffusion behavior observed in intermittent systems (Jou, 1997; Mongiov et al., 2014). The occurrence of a short region of turbulence is a crucial event and the time distance between two consecutive turbulent events, assumed to be of negligible time duration, is given by an inverse power law (IPL) waiting time distribution density with an IPL index μ < 3 and related to fractal-like behavior that can manifest as 1/*f* noise. There are two forms of 1/*f* noise, one with and one without crucial events.

The main purpose of this paper is to contribute to a deeper understanding of these two types of 1/*f*-noise. We do this by proposing the joint use of MSE(*S*_*E*_), Rényi entropy (*H*_α_) and include Diffusion Entropy Analysis (DEA) which is another form of entropy originally introduced to study the complexity of a social process (Scafetta et al., 2001). Note that this was done independently from the work of Costa et al. (2002, 2005) who applied *S*_*E*_ to the study of heartbeat dynamics (Grigolini et al., 2001; Allegrini et al., 2002). The use of these three entropy measures will make it possible to establish which form of 1/*f*-noise is generated by cardiac dynamics and the role played by crucial events.

While a general agreement exists about the importance of 1/*f*-noise for neurophysiological processes, there are two distinct origins of 1/*f*-noise which may be generated by either Gaussian fluctuations, yielding stationary correlation functions, or by non-ergodic fluctuations (Culbreth et al., 2019). The spectrum of 1/*f*-noise can be realized in two ways. The first is through a correlation function which is stationary, but has a diverging correlation time. Such a process generates deviations from ordinary diffusion but without crucial events. The second mechanism results in a non-stationary correlation function generated solely by crucial events. Multiscale analysis cannot distinguish the first from the second mechanism. Diffusion entropy analysis (DEA) has been suitably modified (MDEA), as we subsequently explain, such that MDEA applied in the first case yields ordinary scaling, but in the second case detects anomalous scaling. For this reason, we shall use the modified version of DEA that was recently proposed to assess the empirical nature of 1/*f*-noise (Culbreth et al., 2019). The work of Culbreth et al. (2019) is the original DEA method supplemented by the adoption of stripes, a method already used by Allegrini et al. (2002) that Culbreth et al. (2019) adopted to establish the true nature of 1/*f*-noise. This method is herein applied to the classification of cardiac autonomic neuropathy (CAN) in type 2 diabetes mellitus (T2DM).

We end this introduction by clarifying a confusion regarding 1/*f*-noise that has apparently gone unnoticed in the literature. The confusion stems from the existence of two distinctly different kinds of 1/*f*-noise. There is the fractional Brownian motion (FBM) explanation of 1/*f*-noise first provided by Mandelbrot and Van Ness (1968) and Mandelbrot (1977), which we call Type I. This is separate and distinct from Type II 1/*f*-noise that is generated by crucial events and was first discussed in Allegrini et al. (2002). The confusion arises because the mechanisms generating these two types of noise can appear separately, or they can appear together depending on the complexity of the phenomenon being considered.

**Figure 1:**
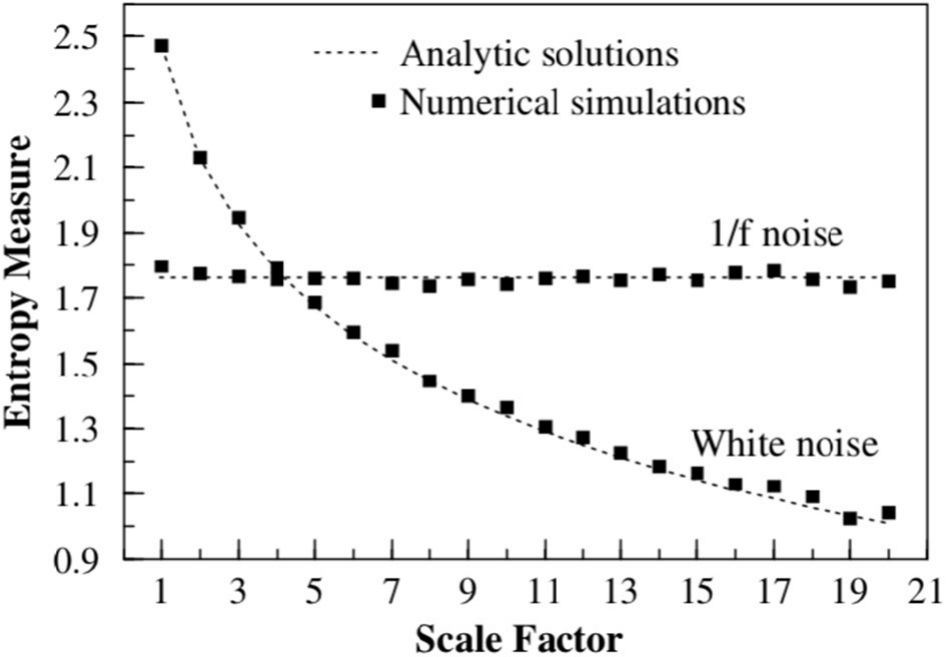
The multiscale entropy (*S*_*E*_) is calculated for two time series, 1/*f* noise and white noise. The time series are both coarse grained by using a scale factor to aggregate the data. The multiscale entropy is graphed vs. the scale factor, where it is seen that 1/*f* noise is independent of the size of the aggregation scale factor, whereas white noise depends strongly on the scaling. This figure is taken from Costa et al. (2002), reprinted here with permission.

**Figure 2:**
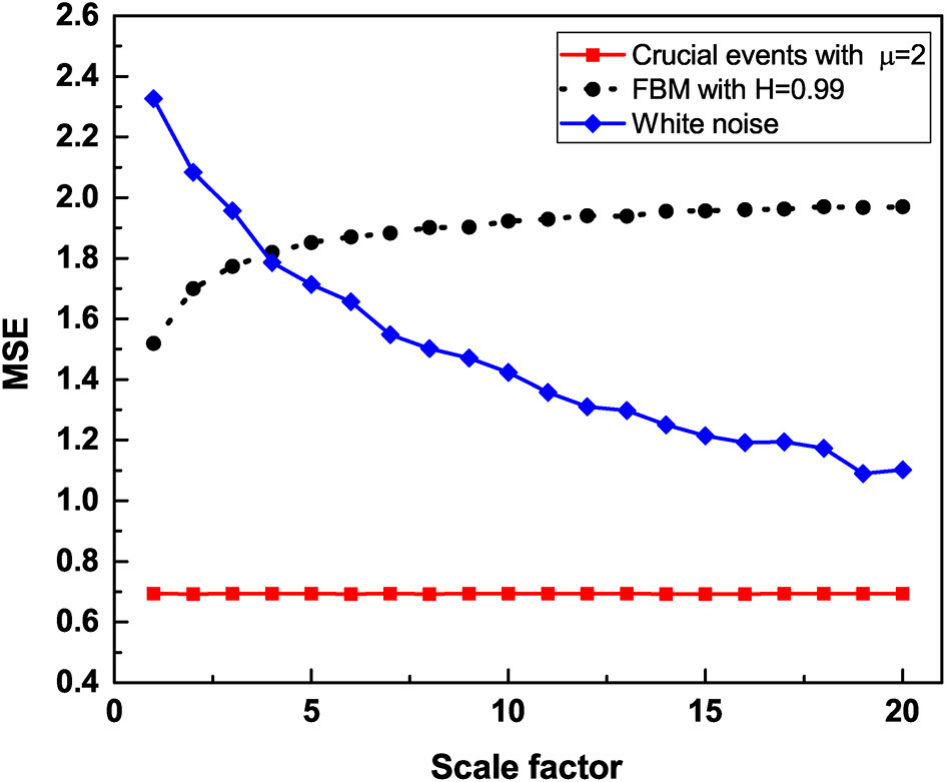
The multiscale entropy (*S*_*E*_) is calculated for three time series, trajectory with crucial events with IPL index μ = 2, FBM with Hurst exponent *H* = 0.99 and white noise. The time series are coarse grained by using a scale factor to aggregate the data. The multiscale entropy is graphed vs. the scale factor. Although both FBM trajectory and trajectory with crucial events generate 1/*f* noise, trajectory with crucial events is independent of the size of the aggregation scale factor while FBM trajectory is independent of the size of the aggregation scale factor only for larger scale factors. White noise depends strongly on the scale.

To facilitate the understanding of the significant results of this paper, Figure 1 displays the Multiscale entropy processing of white noise and Type I 1/*f*-noise, which until recently was the only interpretation of 1/*f*-noise available. Figure 2, on the other hand, compares the Multiscale entropy of white noise with both Type I and II 1/*f*-noise which, as we subsequently show has confused researchers studying heart variability and other complex systems in the past. The black line in Figure 2 illustrates Type I 1/*f*-noise that asymptotically agrees with the traditional FBM 1/*f*-noise depicted in Figure 1. The red line describes Type II 1/*f* noise generated by crucial events and which is remarkably different from the traditional expectation of 1/*f*-noise. The analysis presented here establishes that RR fluctuations host both forms of 1/*f*-noise and that in the definite CAN case only Type II 1/*f*-noise remains. This remarkable conclusion is reached through the proper use of Rényi entropy.

## 2. Materials and Methods

### 2.1. Classification of Cardiac Autonomic Neuropathy

#### 2.1.1. Participant Recruitment

The project was approved by Charles Sturt University Human Research Ethics Committee and conformed with the principles outlined in the Declaration of Helsinki. Participants attended a diabetes health screening clinic in rural Australia and provided written consent following an information session. Type II diabetics and healthy individuals with no known diabetic symptoms, aged 35 and over were selected for this study. All tests were conducted between 10 a.m. and 3 p.m. including ECG recording and cardiac autonomic reflex tests (CARTs) for determination of CAN. Participants had to be free of any diabetic comorbidities and chronic disease including CVD, respiratory disease, or chronic kidney disease. In addition, the health screening included a medication review, acquiring demographic and general health data, retinal, cardiac and foot examination as well as blood and urine biochemistry (Jelinek et al., 2006). Cardiac examination included a 10 s 12-lead ECG, and a 20 min 3-lead (Lead II) recording. From the 20-min RR tachogram, a 15 min segment was selected from the middle in order to remove start up artifacts and movement at the end of the recording. From this shorter recording, the RR intervals were extracted for further HRV analysis including *S*_*E*_, *H*_α_, and DEA. No other information was used in this study.

#### 2.1.2. Cardiac Autonomic Reflex Tests

In order to characterize changes in cardiac neural control mechanisms associated with CAN progression, a battery of five non-invasive cardiovascular reflex tests were applied, including lying to standing, deep breathing, and Valsealva maneuver to identify changes in parasympathetic nervous system control of cardiac rhythm and changes in lying to standing blood pressure and changes in blood pressure associated with hand grip to assess more sympathetic nervous system associated influence on cardiac rhythm (Ewing et al., 1985; Spallone et al., 2011). These tests allowed the cohort to be divided into groups with no cardiac autonomic neuropathy (Normal/Control), early cardiac autonomic neuropathy and definite autonomic neuropathy as outlined in Spallone et al. (2011) and Vinik et al. (2013).

#### 2.1.3. ECG Recording

Electrocardiograms (ECGs) of participants were recorded in a quiet room for a period of 20 min. Three disposable snap electrodes (Brand EL503, BIOPAC System, Inc.) were placed in a Lead II configuration to optimize identification of the QRS fiducial peaks. The ECGs were recorded at a sampling rate set at 400 Hz and notch filter at 50 Hz. These settings were in line with the suggestions of Task Force TFESC (1996). All ECG data were initially manually edited. Ectopic beats were selected visually according to the ECG morphology and were manually deleted. To remove high frequency noise, a 45 Hz low-pass filter was used. In addition, a high pass filter of 3 Hz was applied to all data to adjust for wandering baselines in ECG traces. The RR interval series for each participant was then detrended based on smoothness priors algorithm which does not affect the spectral components significantly, avoiding distortion of data at the end points (Tarvainen et al., 2002).

#### 2.1.4. HRV Analysis

Details of the methodology for both time and frequency domain analysis has been described by Spallone et al. (2011) and Task Force TFESC (1996). Briefly, all HRV features were calculated using custom software on Matlab (Mathworks R2009b) and included SDNN and RMSSD as time domain features. The heart rate signal was decomposed into single spectral components using the Fast Fourier Transform (FFT) with a 50 percent overlap function to construct a spectrum. A Hann window was chosen in order to minimize the discontinuity effect. High frequency (HF), low frequency (LF) power, the ratio (HF/LF), and normalized units were determined as recommended by Task Force TFESC (1996).

### 2.2. Multiscale Entropy

Entropy measures have been applied to linear and non-linear time series and demonstrated to be useful in identifying underlying pathology (Wessel et al., 2000a,b; Voss et al., 2009). ECG time series, however, display multiscale characteristics or are multifractal and can be described by scaling exponents (Ivanov et al., 1999), multiscale entropy (Costa and Healy, 2003), and multiscale Rényi entropy (MsRen) (Cornforth et al., 2014).

Multiscale entropy is based on the concept advocated by Kolmogorov (1965) that a signal should be judged to be totally random if it is proven to be computationally incompressible. The Kolmogorov-Sinai entropy (Pesin, 1976) which is based on the Kolmogorov complexity is applied for the analysis of a time series ξ(*t*) which is defined by:

(1) $$\begin{array}{l} h_{KS}=lim_{N\to \infty }\frac{H_{N}}{N}, \end{array}$$

where the entropy is:

(2) $$\begin{array}{l} H_{N}\equiv -\underset{\xi \left(1\right)...\xi \left(N-1\right)}{\sum }p\;\left(\xi \left(1\right)...\xi \left(N-1\right)\right)\;ln\left[p\;\left(\xi \left(1\right)...\xi \left(N-1\right)\right)\right], \end{array}$$

This has a clear connection with Shannon entropy. However, although attractive, it is computationally challenging and Costa et al. (2002) adopted a coarse-graining procedure leading to results that can be easily understood with the help of Figure 1. According to Costa et al. (2002), the time series under study has to be converted into a coarse grained time series determined by the scale factor τ, where the number of coarse-grained data points is obtained by dividing N by the scale factor. The coarse-grained data points, *y*_*j*_, are calculated from the original data points, *x*_*i*_, according to:

(3) $$\begin{array}{l} y_{j}^{\left(\tau \right)}=\frac{1}{\tau }\overset{j\tau }{\underset{i-\left(j-1\right)\tau +1}{\sum }}x_{i}\;;\;1\leq j\leq \frac{N}{\tau }. \end{array}$$

Consequently, the length of each coarse-grained time series is equal to the length of the original time series divided by the scale factor τ. If the times series under study has a finite correlation time τ_*c*_ when τ < τ_*c*_ this analysis suggests the existence of a structure with some order and records a finite value of *S*_*E*_. However, upon increasing τ, as depicted in Figure 1, the method becomes more sensitive to an increasing randomness thereby yielding smaller and smaller values of *S*_*E*_. In the case of a time series generating 1/*f*-noise the correlation time is divergent, thereby keeping the value of *S*_*E*_ virtually constant. The sample entropy is calculated (Costa et al., 2005) by taking the first *m* data points, calculating their Chebyshev distance to save the “pattern” they make, then counting how many times that pattern occurs in the data set and comparing it to how many times the pattern of length *m* + 1 occurs. The formal calculation is:

(4) $$\begin{array}{l} \text{Sample Entropy}=-\log\left(\frac{A}{B}\right), \end{array}$$

where:

(5) $$\begin{array}{l} A=\text{number of pattern pairs }d\;\left[X_{m+1}\left(i\right),X_{m+1}\left(j\right)\right]<r, \end{array}$$

(6) $$\begin{array}{l} B=\text{number of pattern pairs }d\;\left[X_{m}\left(i\right),X_{m}\left(j\right)\right]<r, \end{array}$$

with *r* being a given “tolerance,” *X* the “pattern,” and *d*[·] representing the Chebyshev distance.

Herein, we seek to reveal the physical origin of processes that lead to *S*_*E*_ being scale independent. We adopt this perspective because the 1/*f*-noise that generates the independence of the scale factor τ is generated in two distinct ways. These distinct types of 1/*f*-noise are herein associated with dramatically different properties, as we show.

The first possibility is based on the stationary correlation function. In fact, if the experimental time series generates a stationary correlation function of the form:

(7) $$\begin{array}{l} \Phi_{\xi }\left(\tau \right)={\left(\frac{T}{T+\tau }\right)}^{\delta }, \end{array}$$

with δ < 1, its correlation function is divergent. T is a constant. It is known (Cakir et al., 2006) that this fluctuation is a generator of fractional Brownian motion (FBM) with the scaling index *H* (also known as Hurst exponent) given by:

(8) $$\begin{array}{l} H=1-\frac{\delta }{2}. \end{array}$$

This is the contribution to Type I 1/*f*-noise. As we have observed earlier, in this case the complexity of the process is generated by the non-integrability of the stationary correlation function. The finite value of T has the twofold purpose of, (a), fitting the normalization constraint of the stationary correlation function of Equation (7), that should be equal to 1 when τ = 0 and, (b), of avoiding non-physical divergence at the origin. The stationary equilibrium correlation function becomes a non-integrable IPL power law for τ tending to infinity. It is important to notice (Lukovic and Grigolini, 2008) that the spectrum *S*(*f*) becomes proportional to 1/*f*^β^ with β = 2*H* − 1, thereby realizing an ideal 1/*f* noise with *H* tending to 1. As we have earlier observed, there exists another alternative that also generates 1/*f*-noise, which is related to criticality, called in this paper Type II 1/*f*-noise.

Contoyiannis et al. (2004) investigated the spontaneous contraction generated by the atria of a frog heart isolated in a physiological solution and on the basis of this observation made the conjecture that interbeat interval (RR) dynamics are an intermittent process generated by a form of self-organization that can be properly described by the Ising model at criticality. This conjecture leading to 1/*f*-noise, requires the adoption of principles associated with turbulence. Accordingly, we can generate the time series ξ(*t*) as follows: there exist extended laminar regions of time duration τ with the waiting-time PDF ψ(τ) given by:

(9) $$\begin{array}{l} \psi \left(\tau \right)\propto \frac{1}{\tau^{\mu }}, \end{array}$$

where the IPL index is within the range:

(10) $$\begin{array}{l} 1<\mu <3. \end{array}$$

The occurrence of an event activates the selection of a new value of τ from the PDF ψ(τ) completely independent of any other inter-event time distances and therefore satisfies the *renewal condition*.

The computational compressibility of these events is established by using the concept of Kolmogorov compressibility and the Kolmogorov-Sinai entropy *h*_*KS*_ (Allegrini et al., 2003), which to a very good approximation can be expressed as Ignaccolo et al. (2001):

(11) $$\begin{array}{l} h_{KS}=z\left(2-z\right)ln2, \end{array}$$

with the relation given by the IPL index:

(12) $$\begin{array}{l} z\equiv \frac{\mu }{\mu -1}. \end{array}$$

When *z* = 1(μ = ∞) the sequence of these fluctuations is totally random (incompressible, according to Kolmogorov), but it becomes more and more compressible with decreasing values of μ, with the surprising property of generating the maximal compressibility at *z* = 2 (μ = 2) where *h*_*KS*_ vanishes. Note that in this theoretical perspective of 1/*f*-noise is based on the key formula for the spectrum (Grigolini et al., 2009):

(13) $$\begin{array}{l} S\left(f\right)\propto \frac{1}{f^{\beta }}, \end{array}$$

with the IPL spectral index:

(14) $$\begin{array}{l} \beta \equiv 3-\mu . \end{array}$$

Grigolini et al. (2009) found that under stress, brain dynamics move from the condition of 1/*f*-noise toward the condition of white noise in accordance with the psychological experiments of Correll (2008). This is also in line with the arguments of phenomenological philosophy of the Dotov group (Dotov et al., 2010, 2017). In fact, the analysis of brain dynamics led investigators (Allegrini et al., 2009) to conclude that healthy physiological functioning of the brain is characterized by the condition of ideal 1/*f*-noise according to Equations (13) and (14) is realized when μ = 2. In the case where crucial events exist and the time series ξ(*t*) is obtained by filling the time region between consecutive crucial events with either the value *W* or the value −*W*, according to the tossing of a fair coin, and consequently the resulting auto-correlation function is not stationary. In the case when the time window of size τ begins with a crucial event, we have:

(15) $$\begin{array}{l} \Phi_{\xi }\left(\tau \right)={\left(\frac{T}{T+\tau }\right)}^{\mu -1}. \end{array}$$

When the window moves along the whole time series, the correlation function becomes infinitely aged and yields:

(16) $$\begin{array}{l} \Phi_{\xi }\left(\tau \right)={\left(\frac{T}{T+\tau }\right)}^{\mu -2}. \end{array}$$

We note that for 2 < μ < 3, the correlation function Equation (16) becomes non-integrable as the correlation function of Equation (7) with δ < 1, although this is due to the aging process of a signal driven by crucial events. In both these cases, the non-integrability generates long-time correlations that are responsible for the scale independence revealed by the adoption of the multiscale entropy. On applying the method of multiscale entropy to both time series, one generated by FBM and the other generated by crucial events, both yield scale independency as observed in Figure 2. It is remarkable that the generator of Type II 1/*f*-noise yields a scale independence even more marked than Type I 1/*f*-noise.

### 2.3. Rényi Entropy

The Rényi entropy (*H*_α_) is a generalization of the Shannon entropy (Rényi, 1960) and is defined as:

(17) $$\begin{array}{l} H\left(\alpha \right)=\frac{1}{1-\alpha }log_{2}\left(\overset{n}{\underset{i}{\sum }}p_{i}^{\alpha }\right). \end{array}$$

where *p*_*i*_ is the probability that a random variable with index i takes a given value out of *n* values, and α is the order of the entropy measure. *H*(0) is the logarithm of n. To address the reliance of entropy methods on bins which introduces an artificial discretisation and leads to a boundary problem, we applied a Gaussian kernel centered on the individual RR interval and all RR intervals are added, weighted by the Gaussian function and based on the distance between the individual RR interval and remaining RR intervals (Cornforth et al., 2014). A density measure can then be calculated for the individual RR interval with index i, as the sum of all contributions from other RR intervals with index j:

(18) $$\begin{array}{l} \rho_{i}=\frac{1}{\sigma \sqrt{2\pi }}\overset{n}{\underset{j=1}{\sum }}\exp^{-\frac{dist_{ij}^{2}}{2\sigma^{2}}} \end{array}$$

where σ is the dispersion of the function and replaces the tolerance as suggested by Costa and Healy (2003). The quantity *dist*_*ij*_ is a distance measure, in this case Euclidean in π dimensions:

(19) $$\begin{array}{l} dist_{ij}=\overset{\pi }{\underset{k=0}{\sum }}{\left(x_{i+k}-x_{j+k}\right)}^{2} \end{array}$$

This yields a probability estimate for each sample of a given length with the desirable property that its value lies between 0 and 1.

### 2.4. Diffusion Entropy

As mentioned earlier, we use the generalized DEA method, denoted MDEA in Culbreth et al. (2019). DEA was originally used by Allegrini et al. (2002) and applied again more recently by Bohara et al. (2017). The signal under study, ξ(*t*), is the RR-time interval, with time *t* being the integer number denoting the beat 1, 2, ..... We divide the ordinate ξ into many bins of size *s* and record the times at which the signal ξ(*t*) moves from one stripe to one of the two nearest neighbor stripes. This is an event which is crucial if it depends on self organized temporal criticality (SOTC) fluctuations (Mahmoodi et al., 2017). The crossing from one stripe to another can also be due to non-crucial fluctuations, being either Poisson or generated by a Gaussian memory process (Culbreth et al., 2019). We create a diffusion process *x*(*t*) with the random walker always jumping ahead by the fixed quantity of 1 when an event, either crucial or not, occurs. This method is an extension of the original work (Allegrini et al., 2002) discussed in depth in the theoretical work (Culbreth et al., 2019). Culbreth et al. (2019) prove that both forms of non-crucial events generate the scaling δ = 0.5, whereas the crucial fluctuations generate anomalous scaling:

(20) $$\begin{array}{l} \delta =\frac{1}{\mu -1}. \end{array}$$

Once the diffusional trajectory *x*(*t*) is created, we convert the single trajectory into many diffusional trajectories using a moving window of size *l*. We record the quantity Δ*x*(*t*) = *x*(*l* + *t*) − *x*(*t*). Considering different values of *t* is equivalent to creating many Gibbs copies that makes it possible to use a probabilistic approach and define the PDF *p*(*x, l*) to evaluate the Shannon entropy:

(21) $$\begin{array}{l} S\left(l\right)=-\int_{-\infty }^{+\infty }p\left(x,l\right)lnp\left(x,l\right). \end{array}$$

The PDF *p*(*x, l*) is expected to be characterized by the scaling structure:

(22) $$\begin{array}{l} p\left(x,l\right)=\frac{1}{l^{\delta }}F\left(\frac{x}{l^{\delta }}\right), \end{array}$$

where *F*(*y*) is a Gaussian function if δ = 0.5 and is a Lévy function with diverging second moment if μ < 3. Since crucial events are defined by Equation (9) with μ < 3, the scaling detected by DEA is larger than the scaling of non-crucial events. We note in fact that due to Equation (20), the condition 2 < μ < 3 yields δ > 0.5. As a consequence, the broadening of *p*(*x, l*) in the long-time limit is expected to be dominated by the crucial scaling of Equation (20). By plugging Equation (22) into Equation (21) we obtain

(23) $$\begin{array}{l} S\left(l\right)=A+\delta lnl, \end{array}$$

where *A* is a constant.

Note that the events detected using the methods of stripes generate both crucial and non-crucial events. The non-crucial events include events generating Type I 1/*f*-noise. Usually the probability ϵ that an event is crucial is very small. The larger ϵ the healthier the physiological process. The evaluation of ϵ is obtained with a procedure illustrated in the earlier work of Bohara et al. (2017) which is based on the observation that the higher the concentration of non-crucial events, the larger is the time *l* necessary for *S*(*l*) to become a linear function of *lnl*. The comments outlined hereby on the meaning of **Figure 4** afford additional information on why the events contributing to Type I 1/*f*- noise may contribute to make ϵ weaker.

## 3. Results

### 3.1. Detecting CAN Progression Using MDEA

#### 3.1.1. Crucial Scaling

**Figure 3:**
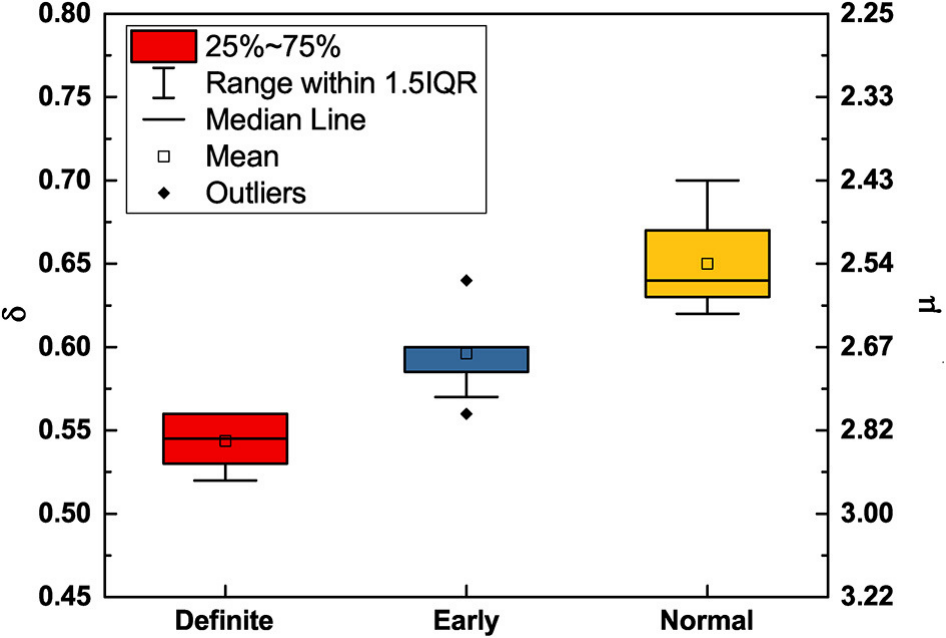
DEA scaling (δ) and complexity index (μ) (obtained from Equation 20) of the HRV time series of participants in different stages of CAN. The mean ($\bar{x}$) and standard deviation (σ) for δ are 0.650,0.027 (normal), 0.596,0.022 (early), and 0.543,0.014 (definite), respectively. Participants in the normal condition have greater complexity (smaller μ, larger δ) compared to those in the definite condition (larger μ, smaller δ).

Using MDEA, Figure 3 illustrates how the scaling parameters δ and μ change with the progression of disease. With disease progression, from the normal to the early and eventually to the definite condition, the scaling δ moves from the healthy super-diffusion scaling toward the scaling δ = 0.5 which corresponds to the normal diffusion of physical systems. In correspondence to this transition from super-diffusion toward a condition close to ordinary diffusion, the IPL index μ moves from values close to μ = 2, to the border between temporary non-stationarity to perennial non-stationarity, at μ = 3, the border between crucial events and Poisson events.

#### 3.1.2. Concentration of Non-crucial Events

**Figure 4:**
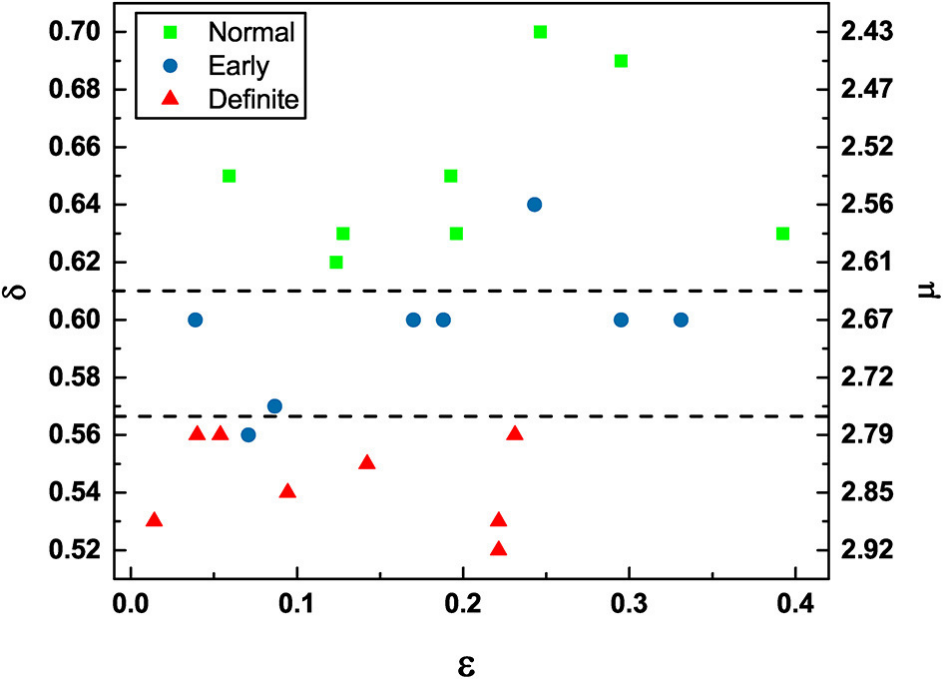
DEA scaling (δ) / complexity index (μ) (obtained from Equation 20) vs. Correlation rate (ϵ) of the HRV time series of participants in different stages of CAN.

The condition μ = 2 is the ideal healthy condition (Allegrini et al., 2002). However, the heartbeat process hosts not only crucial events. The non-crucial events can be either the ordinary Poisson events or events generated by Type I 1/*f*-noise. It is important to stress the existence of this kind of non-crucial events because an important result of this paper is that the definite CAN patients are still characterized by 1/*f*-noise. The non-crucial events generated by type I 1/*f*-noise contribute to increase the concentration on non-crucial events, given by 1 − ϵ. When ϵ = 0, all the events are non-crucial and are a combination of ordinary Poisson events and type I 1/*f*-noise events. When ϵ = 1, all events are crucial. Of course, ϵ = 0 and ϵ = 1 are two ideal conditions and when ϵ < 1, we do not have yet a way of establishing how many of the non-crucial events are type I 1/*f*-noise events and how many of them are Poisson events. We use multiscale entropy to show that the heartbeat process under observation is not white noise but 1/*f*-noise. The fact that increasing values of ϵ are beneficial is proved by Figure 4, which shows that definite CAN patients are characterized by small values of ϵ, while the normal CAN patients seem to move toward the condition of larger values of ϵ.

To understand the significance of the conclusion of this paper that only Type I 1/*f*-noise is left in the definite CAN condition of the processed RR data, we stress that in the original work on crucial events (Allegrini et al., 2002) the non-crucial events were assumed to be Poisson events. In a sequel (Tuladhar et al., 2017) to that early work, it was established that ϵ represents the concentration of all non-crucial events including the Type I 1/*f*-noise (FBM). In fact, Tuladhar et al. (2017) noticed that the RR-trajectory crossings of adjacent stripes signify events characterized by exponential waiting-time PDFs not only in the Poisson case, but more generally by Gaussian processes, including FBM diffusion. This result is based on a theorem presented in Sinn and Keller (2011). We also notice, observing Figure 8 of Tuladhar et al. (2018), that Kundalini Yoga has the effect of increasing μ while also increasing ϵ. Both effects imply that the spectrum becomes whiter, leading us to conclude that we may interpret the practice of Kundalini Yoga as a difficult task sharing this with the increasing severity of autonomic neuropathy (CAN) the property of whitening the spectrum.

#### 3.1.3. Global Perspective

Finally, we can discuss using Figure 4 the global perspective emerging from the results obtained from patients with varying severity of CAN. The division into three groups is to some extent arbitrary. However, we see that the *definite* patients tend to have small values of ϵ and the scaling δ are closer to the border with ordinary diffusion, i.e., δ = 0.5. There is only one *early* patient with δ value in the normal region and only one other *early* patient in the definite region. It is clear that the results recorded in Figure 4 provide strong support for the hypothesis that crucial events are an important signature of healthy physiological function. Moreover, either an excess of non-crucial events (smaller ϵ) or a transition from the healthy condition of μ close to the value of 2 to values close to μ = 3 and beyond is an important signature of disease progression.

### 3.2. Detecting CAN Progression With Multiscale and Rényi Entropy

In this section, we illustrate the benefits of adopting Multiscale Entropy and Rényi Entropy to divide the patients into the normal, early and definite CAN categories, in accordance with Cornforth et al. (2014). The comparison also serves to highlight the additional benefits of the crucial events combined with MDEA in providing a robust pathophysiological model.

#### 3.2.1. Multiscale Entropy

**Figure 5:**
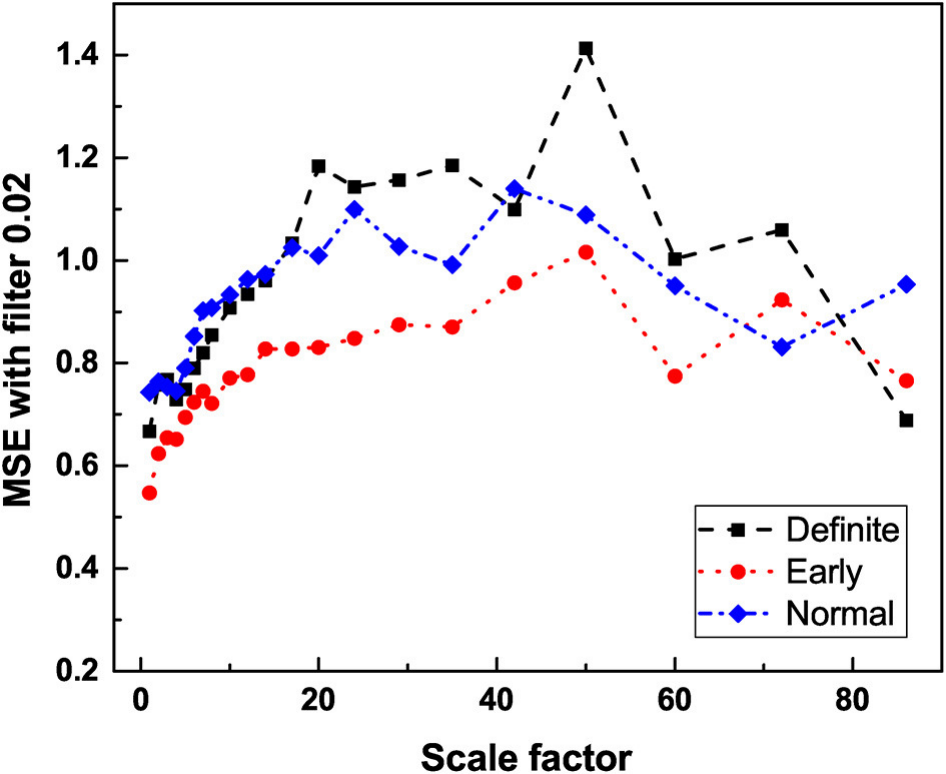
The average entropy measure (*S*_*E*_) vs. scale factor of HRV time series of participants in different stages of CAN using multiscale entropy analysis with filter 0.02. For detailed description of MSE and filter settings see Jelinek et al. (2019).

The result depicted in Figure 5 is consistent with the interpretation that multiscale entropy cannot distinguish 1/*f*-noise generated by crucial events from 1/*f*-noise generated by FBM processes. The level of 1/*f*-noise for definite individuals seems to be higher than for normal individuals. We applied multiscale entropy to surrogate sequences- FBM sequences and crucial event sequences and we found that the level in the first case is higher than in the second. On the basis of this qualitative observation we are inclined to believe the progress of CAN has the effect of turning the 1/*f*-noise generated by crucial events into FBM 1/*f*-noise. Consequently, as the disease progresses the crucial events are lost even though the spectral content of the time series remains unchanged.

#### 3.2.2. Rényi Entropy

**Figure 6:**
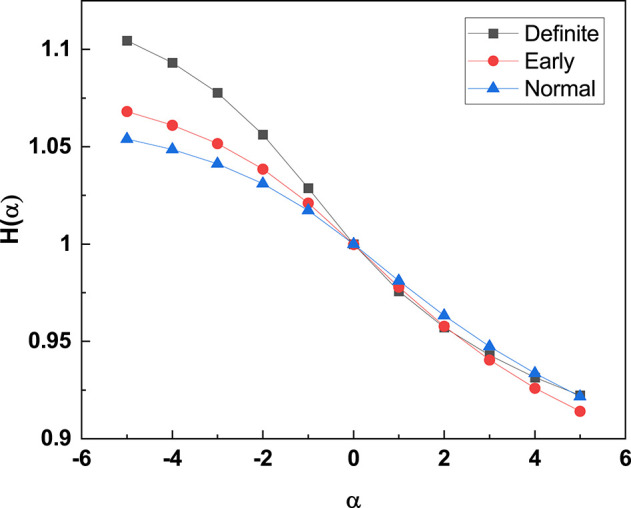
The Rényi entropy [*H*(α)] (with filter 0.02) is calculated for the HRV time series of all participants and the average *H*(α) is plotted for the different stages of CAN. α negatives have the effect of establishing a separation between the three different stages of CAN. For detailed description of Rényi entropy and filter settings see Cornforth et al. (2014).

Note that, according to the authors of Cornforth et al. (2014), the Renyi entropy of Equation (17) with its dependence on α may make it possible to emphasize the importance of events with very small probability. This property of the Rényi entropy allows us to confirm that moving from the normal to the definite CAN condition has the effect of turning the diffusion process generated by the fluctuations of the RR process into a Gaussian process. The PDF *p*(*x, t*) in the normal condition is not Gaussian, but it has slow Lévy tails. Thus, the regions of small probabilities are related to Gaussian tails for the definite category and Lévy tails for the normal category. The weight of the Gaussian tails is much less intense than the weight of the Lévy tails. Thus, the adoption of negative values for α helps illustrate that the definite CAN category has Rényi entropy values significantly larger than the normal and the early CAN categories as well as shown in Figure 6.

The work of Cornforth et al. (2015) shows that in the region of negative α, large values of the Rényi entropy signal that the RR fluctuations become much sharper. This seems to be an indication of a transition from the 1/*f*-noise generated by crucial events, with large variability, to FBM 1/*f*-noise.

## 4. Conclusion

This paper shows that the progression of disease severity makes the crucial event parameter μ move from the border with the condition of perennial non-stationary behavior to the border with Poisson physics, μ = 3. However, this refers to Type II 1/*f*-noise, with β = 3 − μ (Costa and Healy, 2003). This means that Type II 1/f-noise, which is the ideal 1/*f* noise when μ = 2 turns into white noise, β = 0 at μ = 3.

The processing of RR data obtained with MSE shows that heartbeats remain a source of both types of 1/*f*-noise (Type I and II). Figure 5 affords a clear indication of this important property, although the MSE processed data does not afford as clear a way of establishing the difference between the normal and definite CAN conditions as the DEA analysis depicted in Figures 3, 4. Figure 4 shows that the Type I 1/*f*-noise contribution to the 1/*f* spectrum tends to become more important in the definite CAN case, assuming we interpret ϵ as an indicator of Type I 1/*f*- noise as well as of ordinary Poisson processes.

The fact that in the definite CAN case the 1/*f* spectrum is due to FBM is made evident using Rényi entropy, which indicates that the heart rate variability is strongly reduced. We stress that the progress of autonomic neuropathy does not have the effect of whitening the fluctuations as it did in the important work of Guy et al. (2003). The latter work established, with more accurate experimental and theoretical analysis than did the work of Correll (2008), the whitening effects of increasing the difficulty of tasks. The present paper establishes that cardiac dynamics may host both Type I and Type II 1/*f* noise, thereby affording a possible way of explaining the contradiction noticed by the authors of Guy et al. (2003) that in some cases difficult tasks have the effect of increasing rather than decreasing the intensity of the 1/*f*-noise spectrum.

We emphasize that the result of this paper concerning RR time series hosting both Types I and II 1/*f*-noise warrants further investigation. Moreover, we anticipate that identifying the role played by the loss of crucial events in the onset of various pathologies will significantly contribute to the progress of psychological science and clinical medicine.

## Data Availability Statement

The raw data supporting the conclusions of this article will be made available by the authors, without undue reservation.

## Ethics Statement

The studies involving human participants were reviewed and approved by Charles Sturt University Human Research Ethics Committee. The patients/participants provided their written informed consent to participate in this study.

## Author Contributions

HJ conceived and designed the study and obtained the data. GB, RT, GC, and DC did the analysis of the data. HJ, RT, BW, and PG drafted and revised the manuscript. All authors have read and agreed to the published version of the manuscript.

## Conflict of Interest

The authors declare that the research was conducted in the absence of any commercial or financial relationships that could be construed as a potential conflict of interest.
